# Supplementary material for: Association of snR190 snoRNA chaperone with early pre-60S particles is regulated by the RNA helicase Dbp7 in yeast
Source: Nat Commun. 2021 Oct 22;12:6153. doi: 10.1038/s41467-021-26207-w (PMC8536666; doi:10.1038/s41467-021-26207-w)
Supplement: Supplementary file 1 — Supplementary Information [file 41467_2021_26207_MOESM1_ESM.pdf]

SUPPLEMENTARY INFORMATION

Association of snR190 snoRNA chaperone with early pre-60S particles is regulated by the RNA helicase Dbp7 in yeast

Mariam Jaafar, Julia Contreras, Carine Dominique, Sara Martín-Villanueva, Régine Capeyrou, Patrice Vitali, Olga Rodríguez-Galán, Carmen Velasco, Odile Humbert, Nicholas J. Watkins, Eduardo Villalobo, Katherine E. Bohnsack, Markus T. Bohnsack, Yves Henry, Raghida Abou Merhi, Jesús de la Cruz and Anthony K. Henras

SUPPLEMENTARY FIGURES AND TABLES

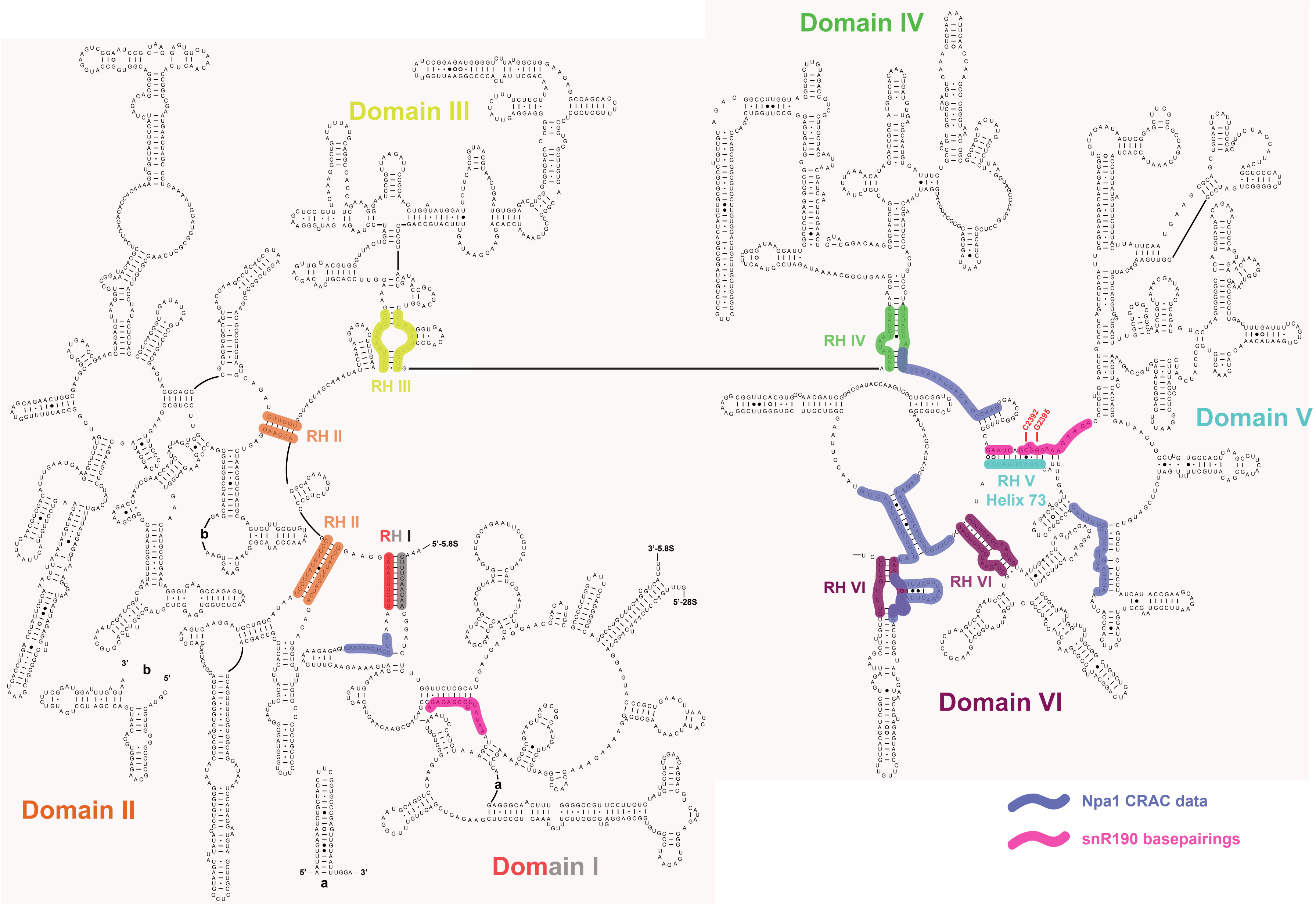

Supplementary Fig. 1 Secondary structure of the 25S rRNA

Secondary structure of the 25S rRNA obtained from (http://www. rna.ccbb.utexas.edu)[1]. Root helices (RH) of the six structural domains of the 25S rRNA (I to VI) are colored (RH I, red/grey; RH II, orange; RH III, yellow; RH IV, green; RH V, light blue/pink; RH VI, purple). Npa1 binding sites in domains I, V and VI are highlighted in blue. 25S rRNA sequences complementary to the antisense boxes A and B of snR190 are highlighted in pink. Position of the predicted methylation site guided by snR190 (G2395) is indicated in red as well as the nucleotide (C2392) found mutated in the rRNA suppressors of the slow-growth of a *dbp7* null mutant described in Fig. 1.

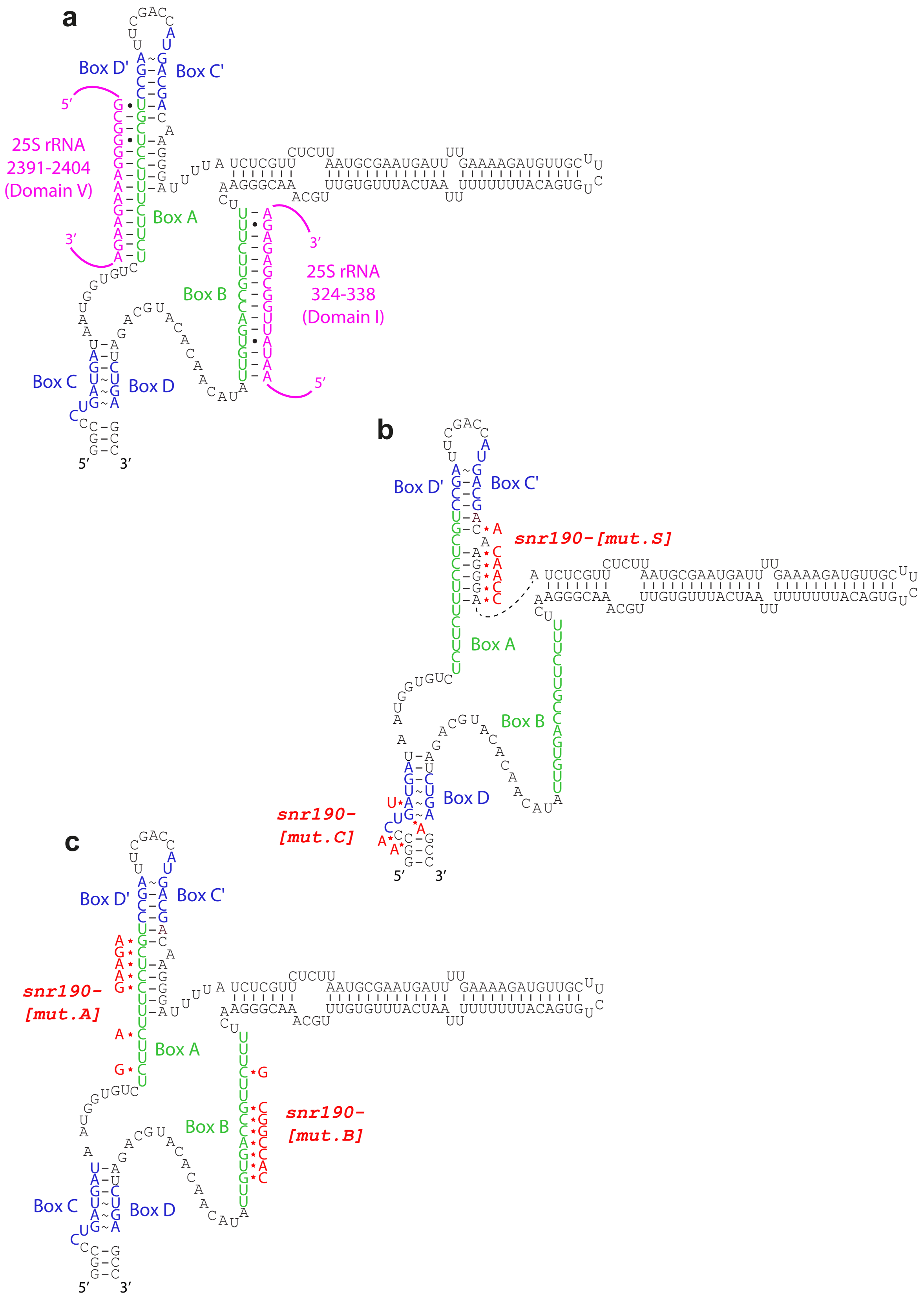

Supplementary Fig. 2 Secondary structure of snR190 snoRNA

a Wild-type snR190 with the regions of interest to this study highlighted in colors: C/D and C'/D' motifs in blue, antisense boxes A and B in green. The 25S rRNA sequences complementary to antisense boxes A and B of snR190 are colored in pink (these sequences are also highlighted in pink on the 2D structure of the 25S rRNA presented in Supplementary Fig. 1). **b** Secondary structure of snR190 with mutations (in red) introduced by CRISPR-Cas9 in box C and terminal stem to abrogate expression of the snoRNA (*snr190-[mut.C]*) as well as the mutations introduced independently to disrupt the internal base-pairing involving part of the antisense box A (*snr190-[mut.S]*). **c** Secondary structure of snR190 with mutations (in pink) introduced in box A (*snr190-[mut.A]*), box B (*snr190-[mut.B]*) or both (*snr190-[mut.AB]*) to disrupt their base-pairing with 25S rRNA sequences.

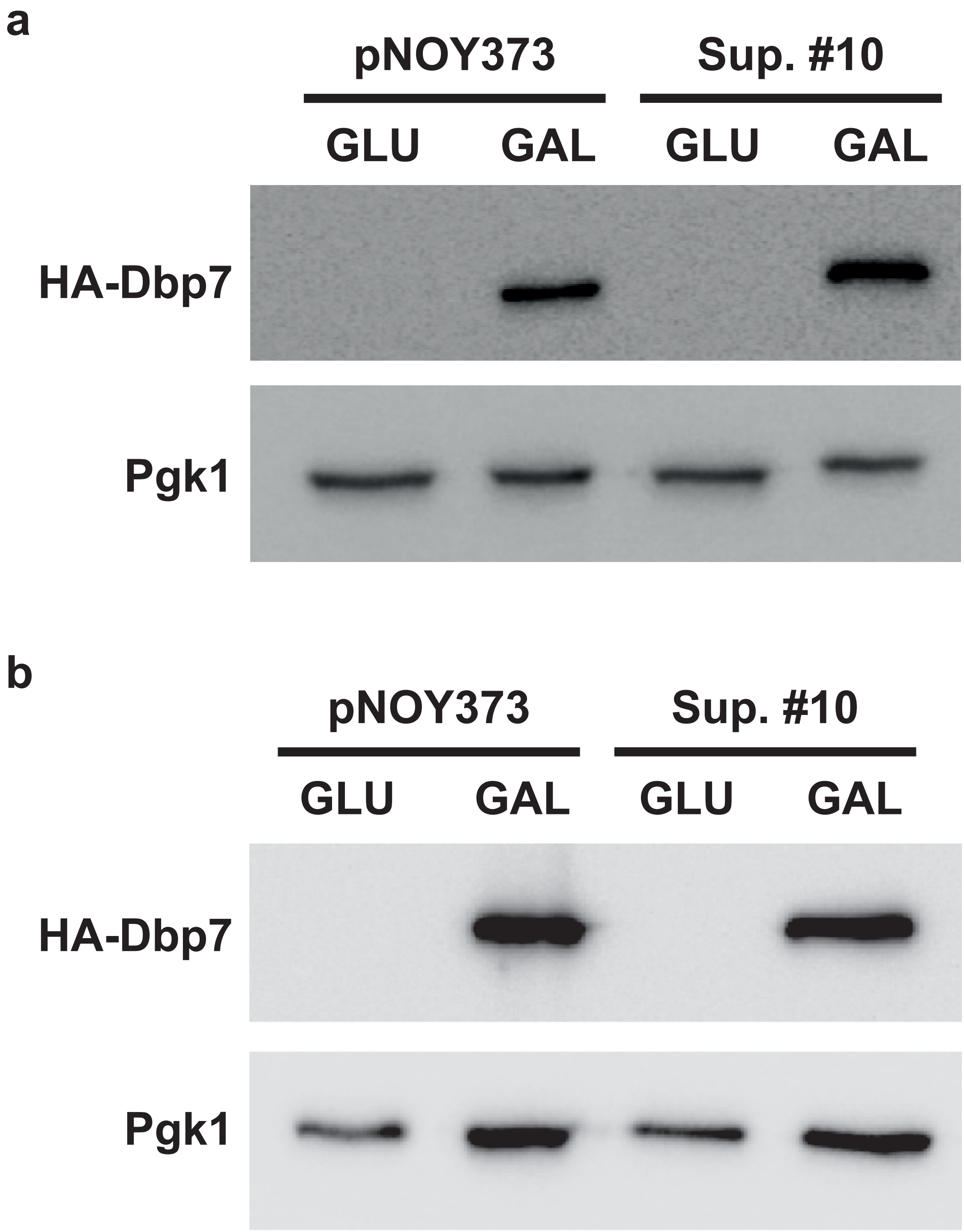

Supplementary Fig. 3 Expression levels of HA-Dbp7 in the control and Sup. #10 strains

a, **b** The EMY65 strain transformed with either the control pNOY373 plasmid or the suppressor Sup. #10 plasmid were grown in SGal (GAL) or SD (GLU) liquid media. Cell extracts were prepared and assayed by western blotting. Equal amount of total protein (40 µg) were loaded in each lane. HA-Dbp7 and Pgk1 were revealed using anti-HA and anti-Pgk1 antibodies, respectively. Two biological replicates are presented (**a**, **b**).

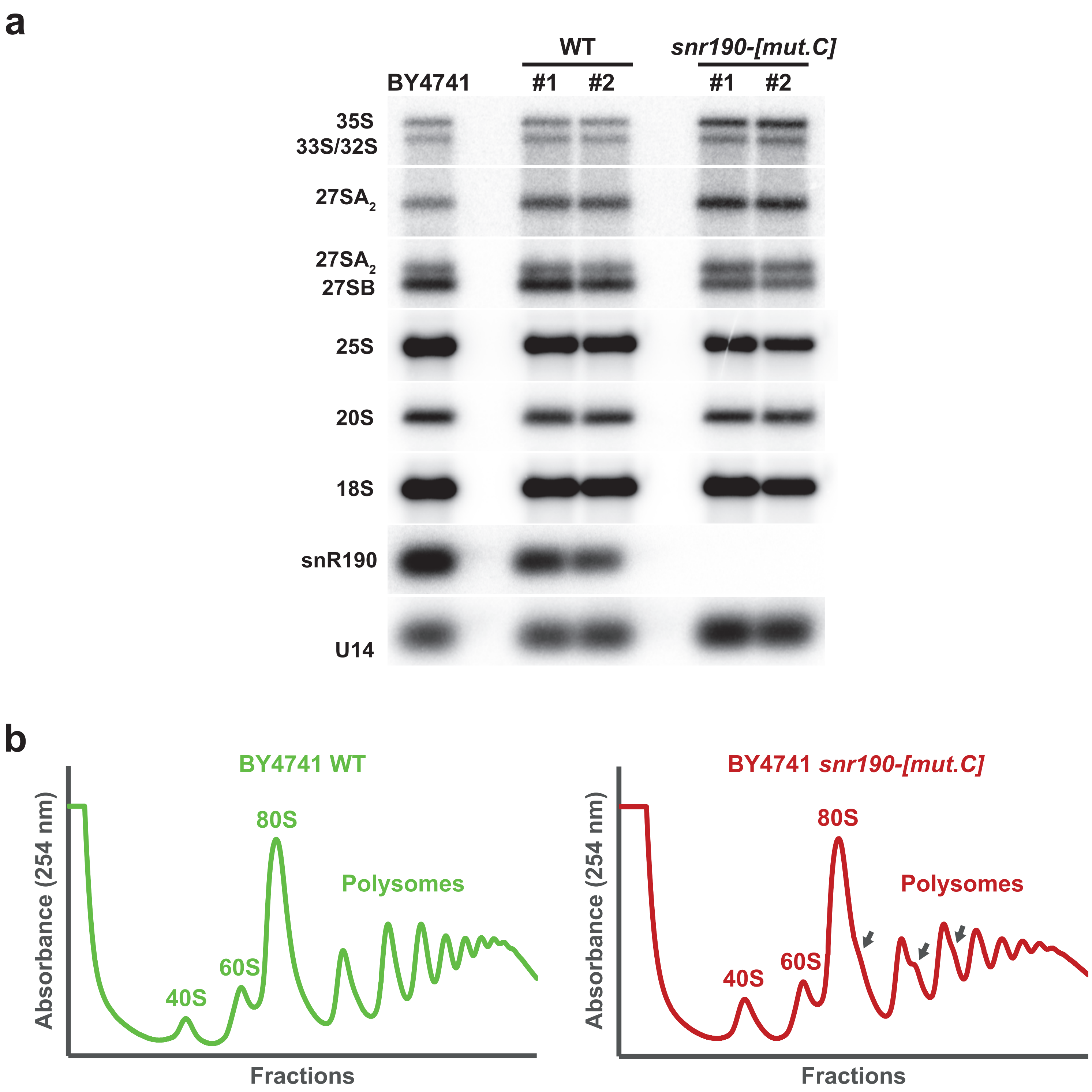

Supplementary Fig. 4 snR190 is required for optimal maturation of pre-60S r-particles

a Accumulation levels of rRNA precursors in isogenic wild-type and *snr190-[mut.C]* strains in the BY4741 background. Total RNA extracted from two independent clones (#1 and #2) for each strain was analyzed by northern blotting. The different RNA molecules, indicated on the left of each panel, were detected using specific radiolabeled probes (Supplementary Table 4). **b** Polysome profiles of BY4741 wild-type (green) and *snr190-[mut.C]* (red) strains. Total cellular extracts prepared from these strains were centrifuged through 10% to 50% sucrose gradients. A_{254} was measured during gradient fractionation. The identity of the different peaks is indicated above and half-mers are marked with arrows.

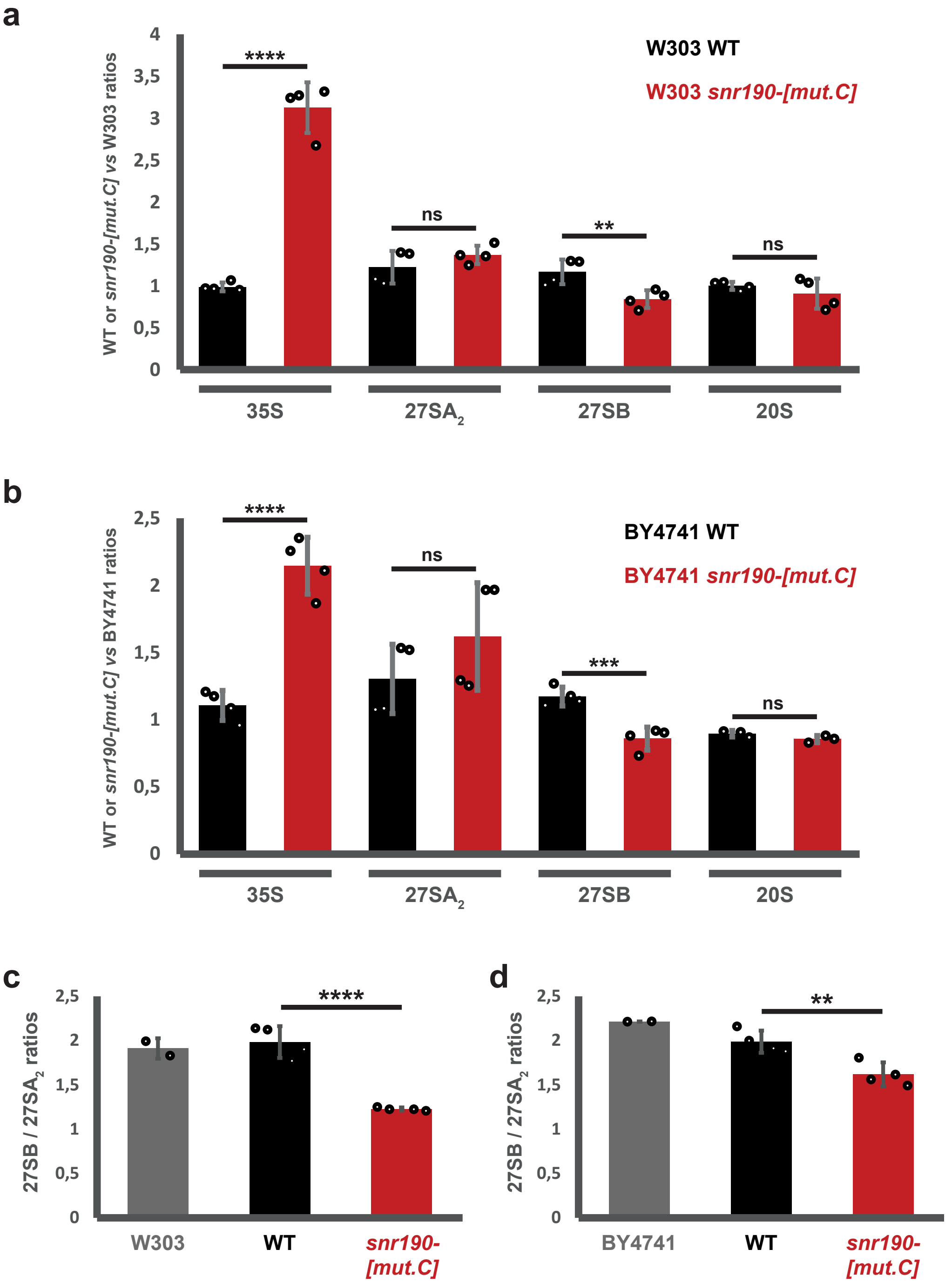

Supplementary Fig. 5 Quantification of the pre-rRNA processing defects in the absence of snR190

a Quantification of rRNA precursor levels in the wild-type (WT) or *snr190-[mut.C]* strains in the W303 background. Radioactive signals presented in Fig. 2a were quantified from PhosphorImager images using MultiGauge software. The histogram represents the ratios of the indicated precursor levels in the wild-type (WT, black) or *snr190-[mut.C]* (red) strains with respect to the levels of the same precursors in the isogenic W303 strain. Data correspond to two biological replicates analyzed as two technical replicates. Histograms represent the mean values +/- standard deviation. The individual data points are shown. Statistically significant differences determined using one-tailed Student's t-test are indicated by asterisks (35S: ****= p<0.0001 (0.00001); 27SA$_2$: ns= not significant; 27SB: **= p<0.01 (0.005827); 20S: ns= not significant). **b** Quantification of rRNA precursor levels in the wild-type (WT) or *snr190-[mut.C]* strains in the BY4741 background. The signals presented in Supplementary Fig. 4a were quantified from PhosphorImager images using MultiGauge software. The histogram represents the ratios of the indicated precursor levels in the wild-type (WT, black) or *snr190-[mut.C]* (red) strains with respect to the levels of the same precursors in the isogenic BY4741 strain. Data correspond to two biological replicates analyzed as two technical replicates. Histograms represent the mean values +/- standard deviation. The individual data points are shown. Statistically significant differences determined using one-tailed Student's t-test are indicated by asterisks (35S: ****= p<0.0001 (0.000063); 27SA$_2$: ns= not significant; 27SB: ***= p<0.001 (0.000656); 20S: ns= not significant). **c** Quantification of the 27SB/27SA$_2$ ratios in the W303 (grey), WT (black) and *snr190-[mut.C]* (red) strains, from the precursor levels measured in (**a**), corresponding to the northern blot presented in Fig. 2a. Data correspond to one (W303) or two biological replicates (WT, *snr190-[mut.C]*) analyzed as two technical replicates. Histograms represent the mean values +/- standard deviation. The individual data points are shown. Statistically significant differences determined using one-tailed Student's t-test are indicated by asterisks (****= p<0.0001 (0.000078)). **d** Quantification of the 27SB/27SA$_2$ ratios in the BY4741 (grey), WT (black) and *snr190-[mut.C]* (red) strains, from the precursor levels measured in (**b**), corresponding to the northern blot presented in Supplementary Fig. 4a. Data correspond to two biological replicates analyzed as two technical replicates. Histograms represent the mean values +/- standard deviation. The individual data points are shown. Statistically significant differences determined using one-tailed Student's t-test are indicated by asterisks (**= p<0.01 (0.003581)).

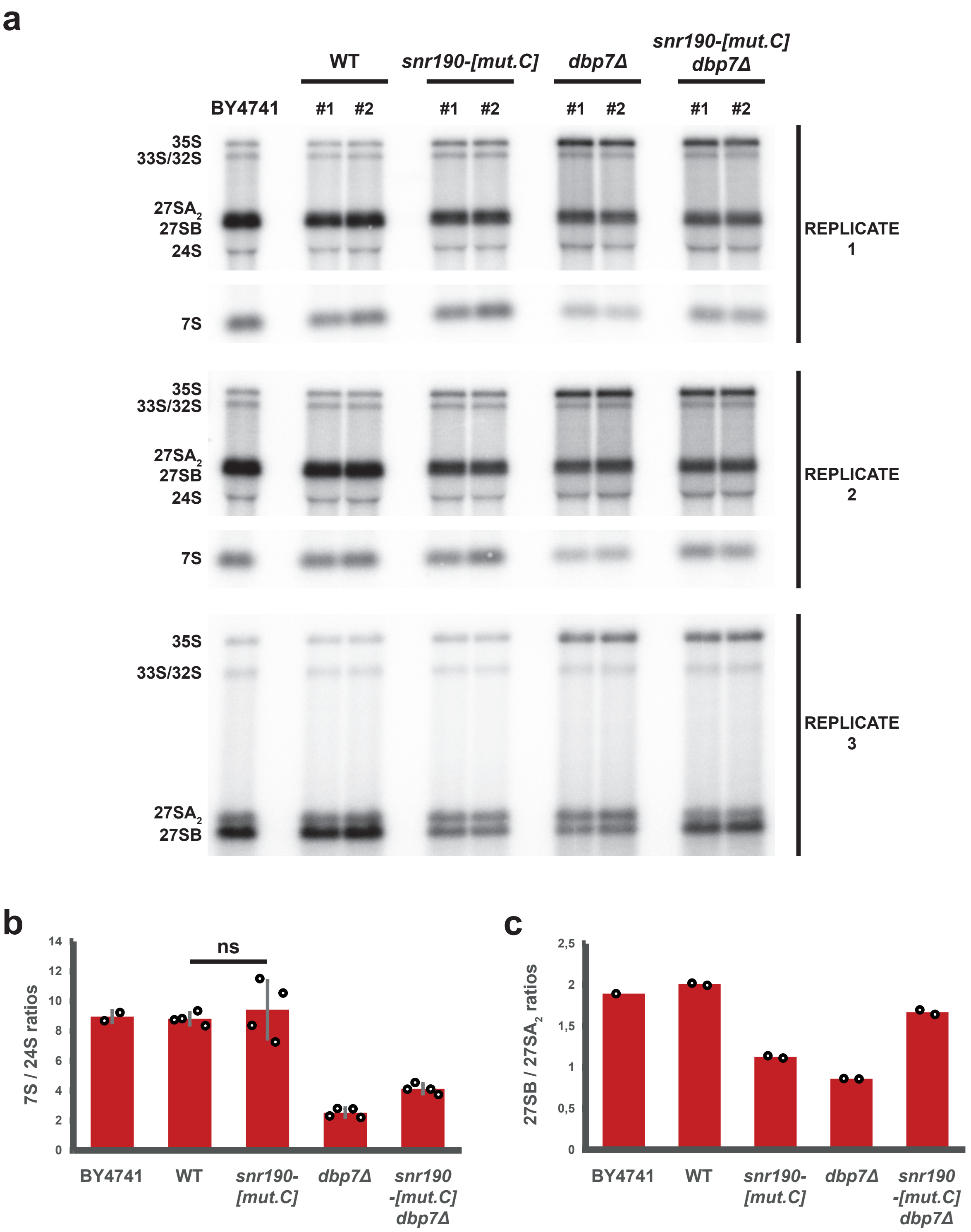

Supplementary Fig. 6 Comparative analysis of pre-rRNA processing in wild-type and mutant strains

a Accumulation levels of rRNA precursors in isogenic wild-type (WT), *snr190-[mut.C]*, *dbp7Δ*, and *snr190-[mut.C] dbp7Δ* strains in the BY4741 background. Total RNA extracted from two independent clones (#1 and #2) for each strain was analyzed by northern blotting. The different RNA molecules, indicated on the left of each panel, were detected using specific radiolabeled probes (Supplementary Table 4). Three technical replicates are shown. Replicates 1 and 2 differ from replicate 3 in term of electrophoresis time. Electrophoresis time was short in replicates 1 and 2 to allow detection of the 7S precursor to the 5.8S rRNA. Electrophoresis time was longer in replicate 3 to separate the $27SA_2$ and 27SB intermediates to allow accurate quantifications. **b** Quantification of the 7S/24S ratios for the indicated strains from replicates 1 and 2 in (**a**). The 24S pre-rRNA[2] was chosen as an invariant species for unbiased quantification. Data correspond to one (BY4741) or two (all the other strains) biological replicates analyzed as two technical replicates (replicates 1 and 2 in **a**). Histograms represent the mean values +/- standard deviation. The individual data points are shown. Statistically significant differences determined using one-tailed Student's t-test (ns= not significant). **c** Quantification of the $27SB/27SA_2$ ratios for the indicated strains from replicate 3 in (**a**). The individual data points are shown.

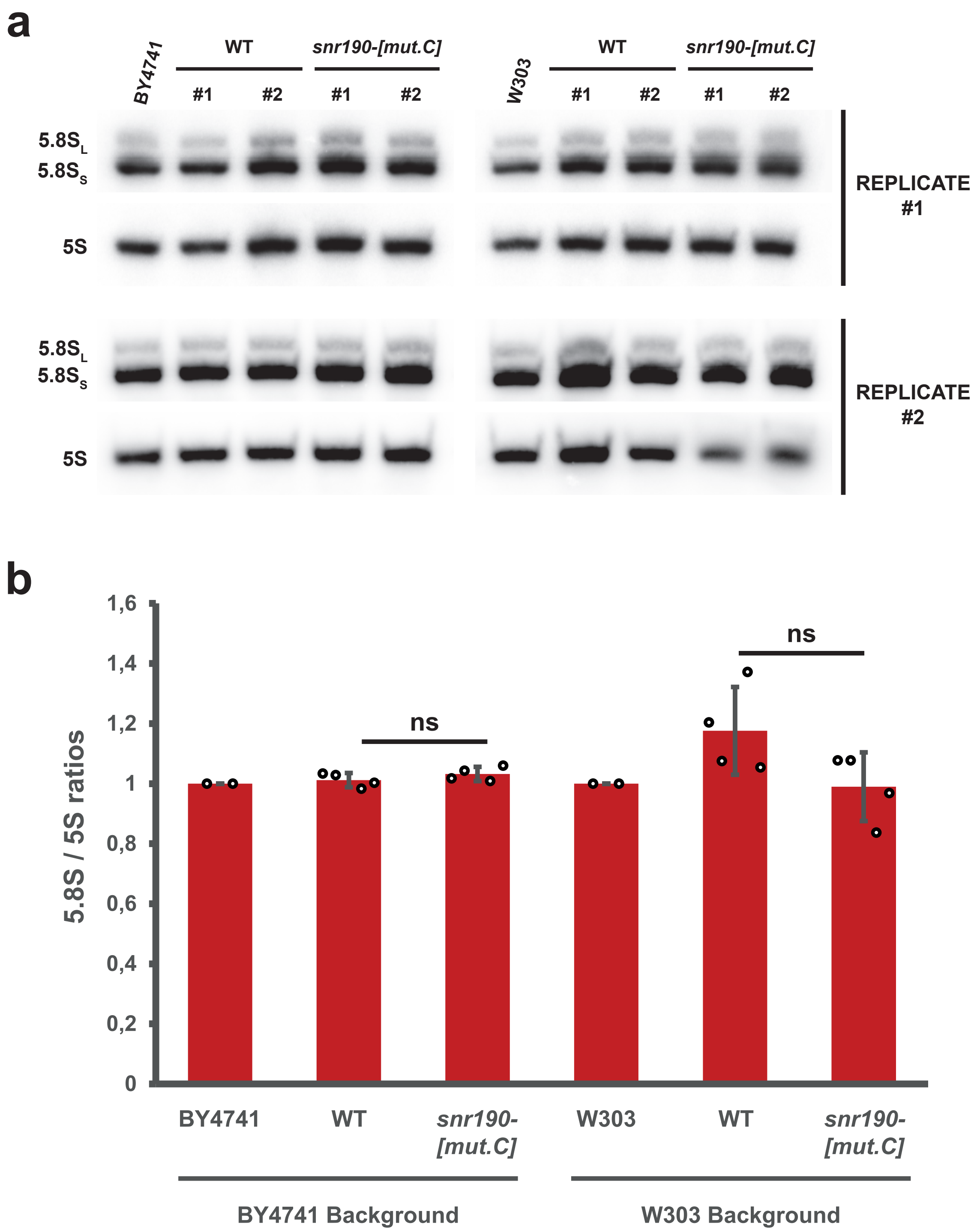

Supplementary Fig. 7 Loss-of-function of snR190 does not affect mature 5.8S levels

a Accumulation levels of 5.8S and 5S rRNAs in isogenic wild-type (WT) and *snr190-[mut.C]* in the BY4741 and W303 backgrounds. Total RNA extracted from two independent clones (#1 and #2) for each strain was analyzed by northern blotting. The 5.8S and 5S rRNAs were detected using specific radiolabeled probes (Supplementary Table 4). Two technical replicates are shown. **b** Quantification of the 5.8S/5S ratios for the indicated strains. Data correspond to one (BY4741 and W303) or two (WT and *snr190-[mut.C]*) biological replicates analyzed as two technical replicates (replicates 1 and 2 in **a**). Histograms represent the mean values +/- standard deviation. The individual data points are shown. Statistically significant differences determined using one-tailed Student's t-test are indicated (ns= not significant).

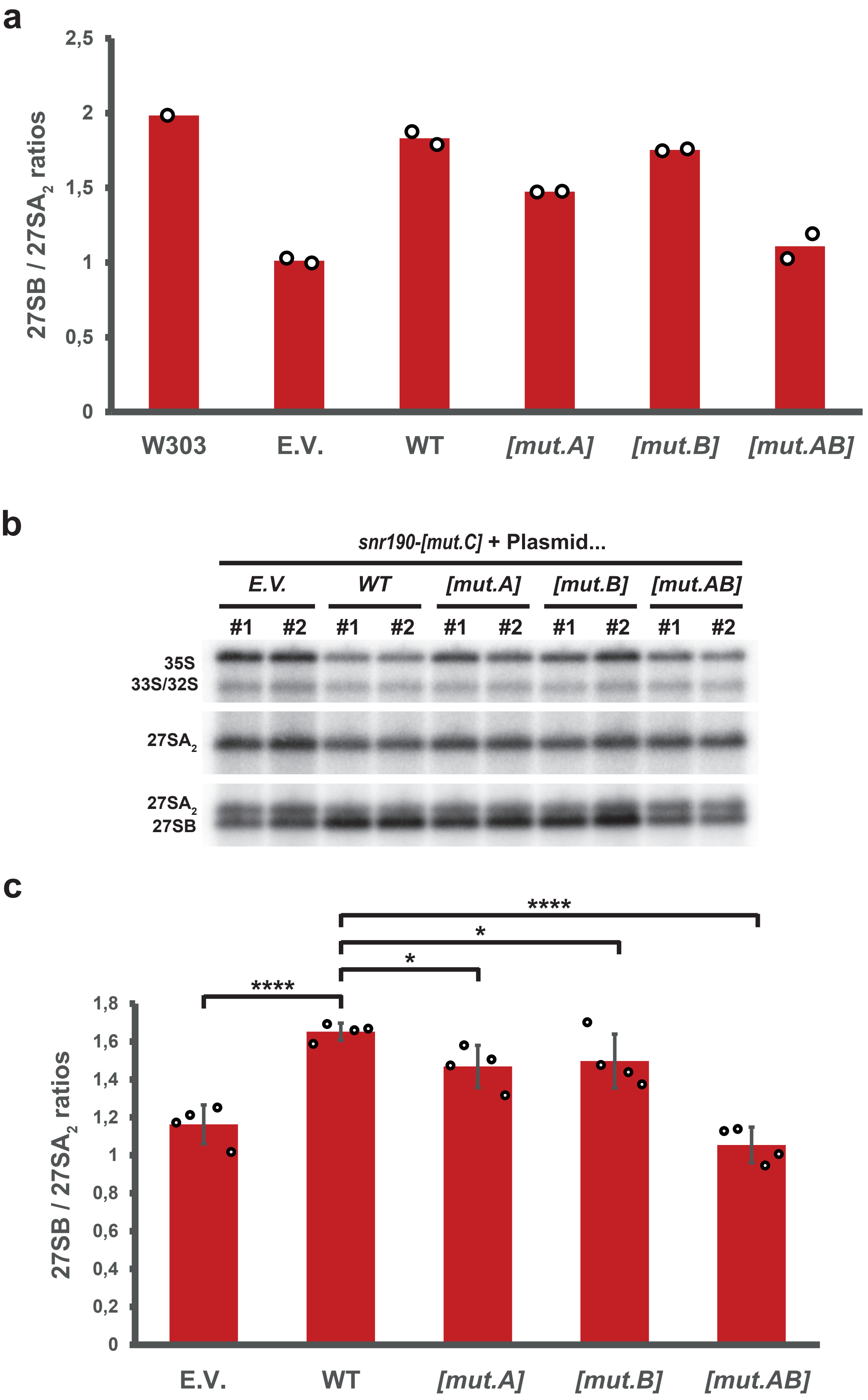

Supplementary Fig. 8 Antisense boxes A and B of snR190 are required for its function in LSU synthesis

a Quantification of the northern blot signals obtained in Fig. 4b using PhosphorImager data and Multi-Gauge software. The histogram represents the mean ratios of the $27SA_2$ over 27SB signals for the indicated strains. Individual data points are shown. **b** Accumulation levels of rRNA precursors in the wild-type BY4741 strain or in the *snr190-[mut.C]* strain transformed with plasmids supporting expression of wild-type snR190 (WT) or bearing mutations in its antisense elements box A (*snr190-[mut.A]*), box B (*snr190-[mut.B]*) or both (*snr190-[mut.AB]*), or with the empty vector as control (E.V.). Experiments were performed as explained in the legend of Fig. 2b. c Quantification of the $27SB/27SA_2$ ratios for the indicated strains from the precursor levels measured in (**b**). Data correspond to two biological replicates analyzed as two technical replicates. Histograms represent the mean values +/- standard deviation. The individual data points are shown. Statistically significant differences determined using one-tailed Student's t-test are indicated by asterisks (E.V. *vs* WT: ****= $p<0.0001$ (0.000063); WT *vs [mut.A]*: *= $p<0.05$ (0.011137); WT *vs [mut.B]*: *= $p<0.05$ (0.04189); WT *vs [mut.AB]*: ****= $p<0.0001$ (0.000013)).

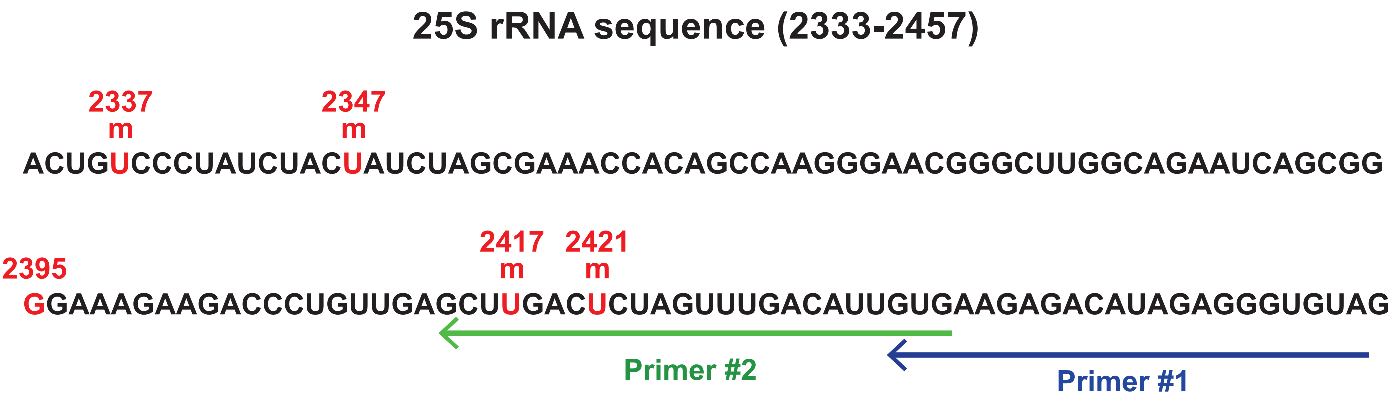

Supplementary Fig. 9 Position of primers used to map 25S rRNA G2395 putative methylation

Sequence of the 25S rRNA surrounding guanosine G2395 predicted to be methylated by snR190. The neighboring methylated nucleosides, experimentally identified in Fig. 3 are indicated in red. Position of the two primers (Primer #1, distal, blue; Primer #2, proximal, green) used in reverse transcription experiments in Fig. 3 are also indicated.

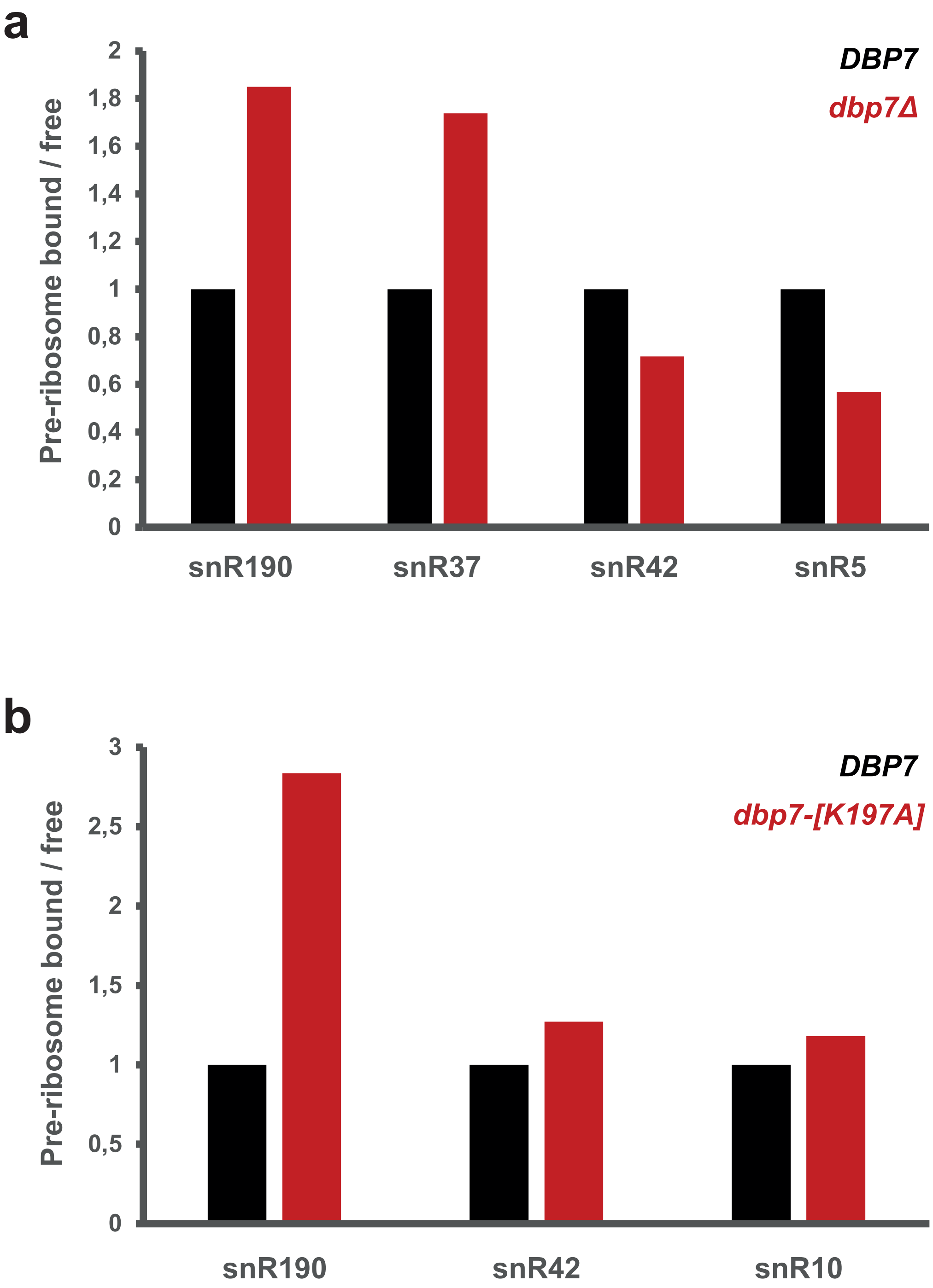

Supplementary Fig. 10 The ATPase activity of Dbp7 is required for snR190 release from pre-ribosomal particles

a Quantification of the sedimentation profiles of different snoRNAs in wild-type or *dbp7Δ* strains (Fig. 5c). For each snoRNA, the radioactive signals detected in the free fractions (1-5) of the gradients or in the fractions containing pre-ribosomes (7-18) were quantified using PhosphorImager data and MultiGauge software. The histogram represents pre-ribosome-bound versus free signal ratios for the indicated snoRNA in the wild-type (*DBP7*, black) or *dbp7Δ* (red) strains. **b** Quantification of the sedimentation profiles of different snoRNAs in cells expressing $Dbp7_{WT}$ or $Dbp7_{K197A}$ (Fig. 6c). For each snoRNA, the radioactive signals detected in the free fractions (1-3) of the gradients or in the fractions containing pre-ribosomes (7-13) were quantified using PhosphorImager data and MultiGauge software. The histogram represents pre-ribosome-bound versus free signal ratios for the indicated snoRNAs in cells expressing $Dbp7_{WT}$ (*DBP7*, black) or $Dbp7_{K197A}$ (red).

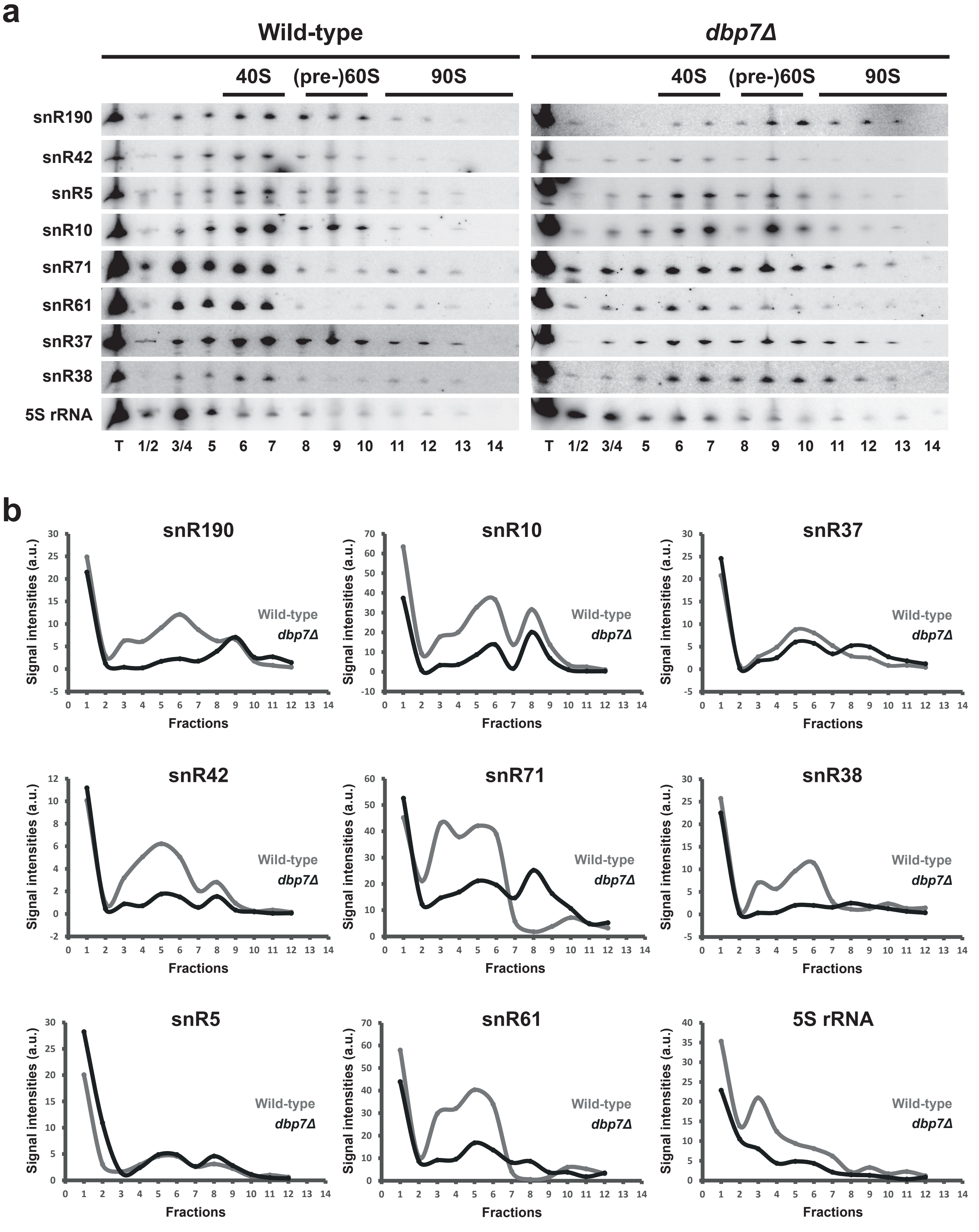

Supplementary Fig. 11 Dbp7 is required for snR190 release from pre-ribosomal particles

a Sedimentation profile of snoRNAs in wild-type or *dbp7Δ* strains. Total cellular extracts were centrifuged through 7 % to 50 % low-Mg^{2+} sucrose gradients. Absorbance profiles at A_{254} were measured during gradient fractionation. The approximate positions of the peaks corresponding to the 40S, (pre-)60S and 90S particles are indicated on the basis of previous experiments[3,4]. RNAs extracted from the first 14 fractions were analyzed by northern blotting to detect the indicated snoRNAs. **b** Quantification of the snoRNA sedimentation profiles observed in (**a**). For each snoRNA, the radioactive signals detected in each fraction was plotted against fraction numbers.

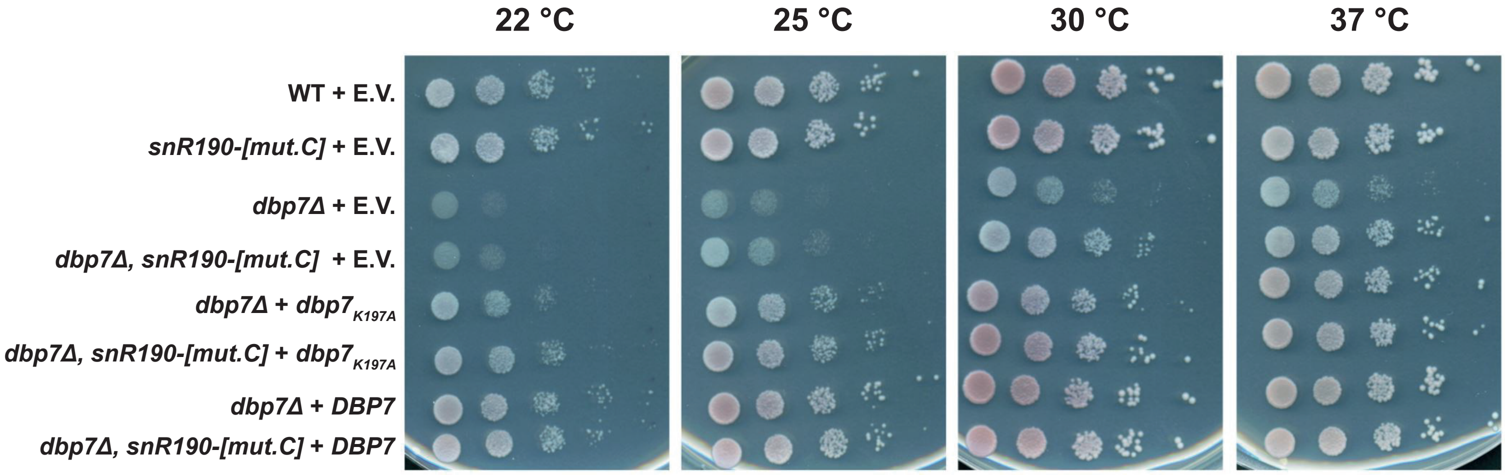

Supplementary Fig. 12 Loss-of-function of snR190 alleviates the growth defect of cells expressing Dbp7$_{K197A}$

The wild-type W303 strain (WT) and the *snR190-[mut.C]*, *dbp7Δ* and *dbp7Δ snR190-[mut.C]* mutant strains were transformed with plasmids expressing Dbp7$_{WT}$ or Dbp7$_{K197A}$, or the empty vector (E.V.) as control, as indicated on the figure. Serial dilutions of the transformants were spotted on SD medium and plates were incubated for 3 days at the indicated temperatures.

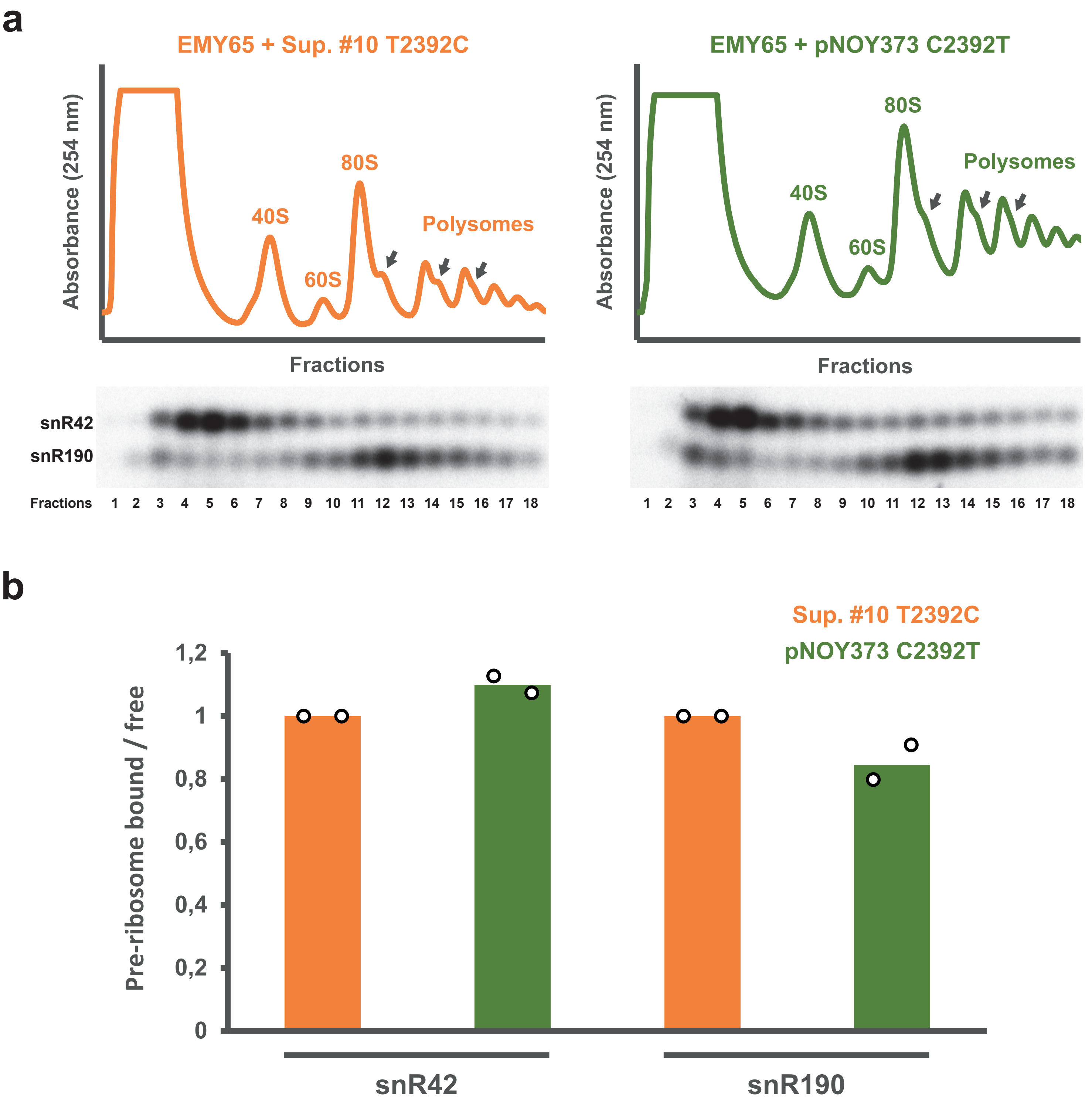

Supplementary Fig. 13 25S rRNA mutation C2392U partially alleviates retention of snR190 in pre-ribosomal particles

a Strain EMY65 was transformed with either the Sup. #10 plasmid in which the suppressor mutation (T2392) has been reverted to the wild-type cytosine (T2392C, orange) or with the pNOY373 plasmid in which the suppressor C2392T mutation has been specifically introduced (C2392T, green). Strains were grown in SD medium and total cellular extracts prepared from these strains were centrifuged through 10% to 50% sucrose gradients. A_{254} was measured during gradient fractionation. The identity of the different peaks is indicated above and half-mers are marked with arrows. RNAs extracted from the first 18 fractions were analyzed by Northern blotting to detect snR190 and snR42 snoRNAs. **b** Quantification of the sedimentation profiles of snR190 and snR42 snoRNAs in the strains described in (**a**). For each snoRNA, the radioactive signals detected in the free fractions (1-5) of the gradients or in the fractions containing pre-ribosomes (7-18) were quantified using PhosphorImager data and MultiGauge software. The histogram represents the mean pre-ribosome-bound versus free signal ratios for the indicated snoRNAs in strain EMY65 containing plasmid Sup. #10 T2392C (orange) or pNOY373 C2392T (green). The individual data points are shown.

a

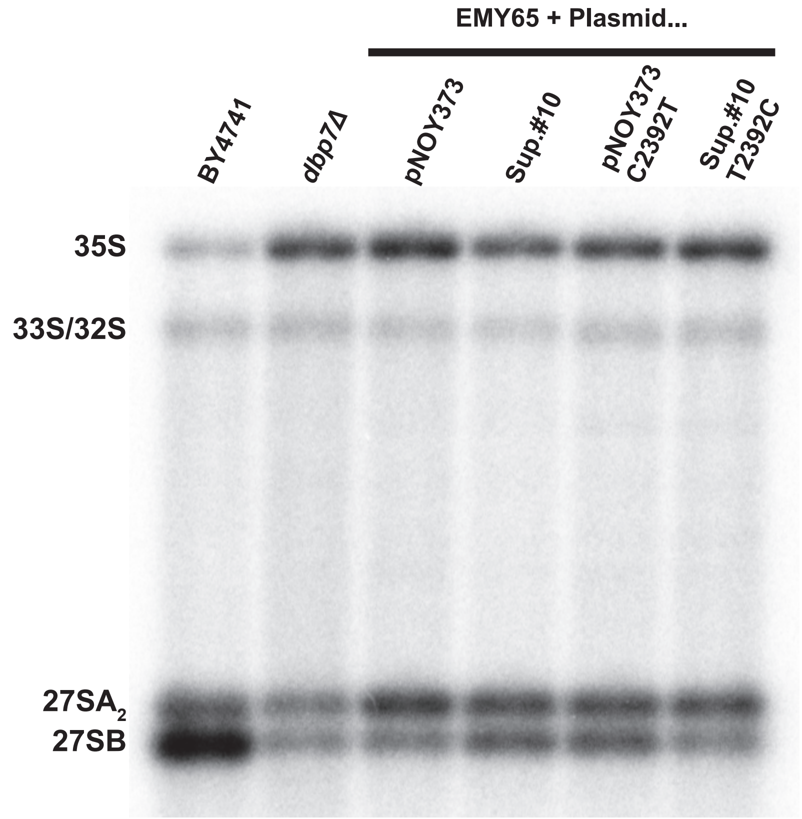

b

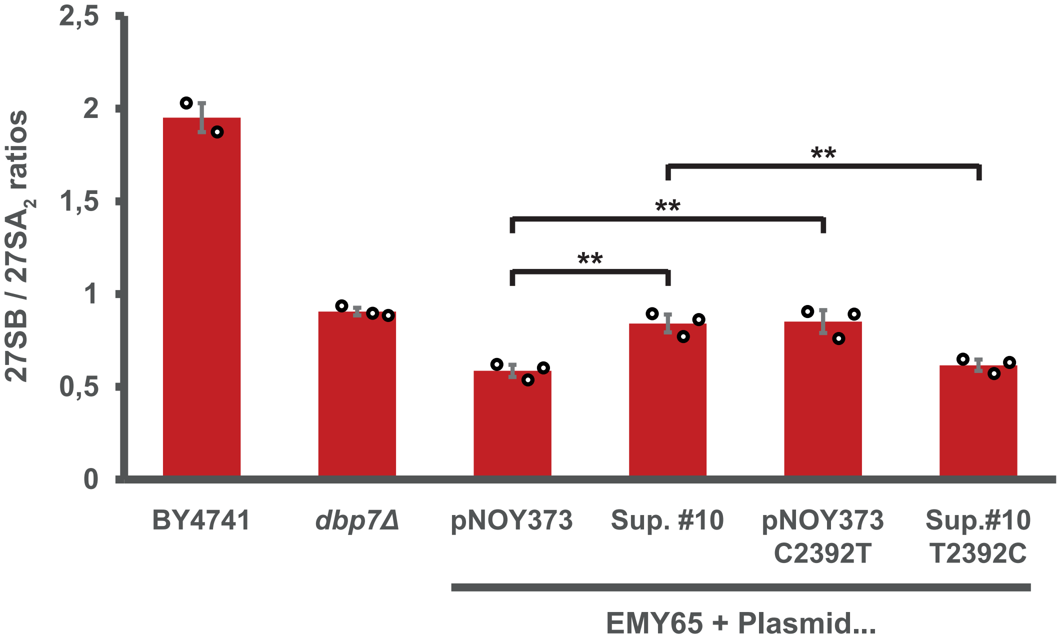

Supplementary Fig. 14 Impact of 25S rRNA mutation C2392U on pre-rRNA processing

a Accumulation levels of rRNA precursors in isogenic wild-type (WT) and *dbp7 Δ* strains in the BY4741 background, as well as in EMY65 strain transformed with the indicated plasmids and grown on SD medium. Total RNA extracted from these strains was analyzed by northern blotting. The different RNA molecules, indicated on the left of each panel, were detected using specific radiolabeled probes (Supplementary Table 4). **b** Quantification of the 27SB/27SA$_2$ ratios for the indicated strain using PhosphorImager data (**a**) and MultiGauge software. Data correspond to two (BY4741) or three (all the other strains) technical replicates. Histograms represent the mean values +/- standard deviation. The individual data points are shown. Statistically significant differences determined using one-tailed Student's t-test are indicated by asterisks (pNOY373 *vs* Sup. #10: **= $p<0.01$ (0.002335); pNOY373 *vs* pNOY373 C2392T: **= $p<0.01$ (0.003574); Sup. #10 *vs* Sup. #10 T2392C: **= $p<0.01$ (0.003384)).

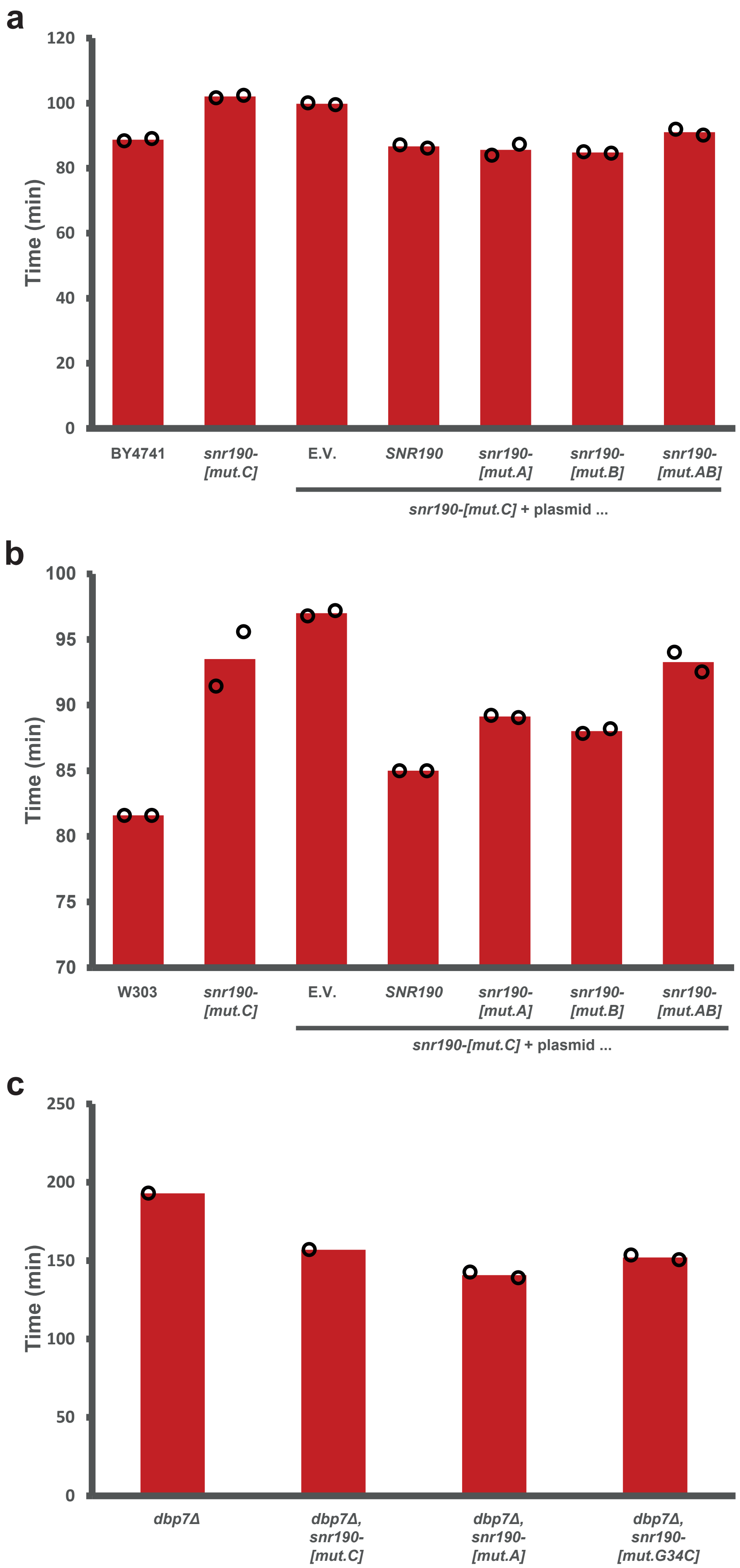

Supplementary Fig. 15 Doubling time of yeast strain analyzed in this study displayed as bar charts

a, **b**, **c** The indicated strains were grown under exponential conditions in YPD medium supplemented or not with G418 depending on the presence of pCH32 plasmid derivatives (G418 resistance marker). Turbidity at 600 nm was measured every two hours and the values were plotted over time in Excel (2016) with a logarithmic scale on the y axis. Doubling times were calculated according to the slopes of the curves. Histograms represent the mean values calculated out of two independent experiments for all strains except the *dbp7Δ*, and *dbp7Δ snr190-[mut.C]* strains (one replicate). See Supplementary Table 2 for a summary.

Suppressor #2 mutations		
Position	Domain	Mutation
912	II (ES10)	Transition (G→A)
1078-1079	II (ES12)	Insertion (A)
1101-1102	II (ES12)	Insertion (A)
1112-1113	II (ES12)	Insertion (GA)
1907-1908	V (H62)	Insertion (A)
2367	V (H72)	Deletion (A→Δ)
2392	V (H73)	Transition (C→U)

Suppressor #10 mutations		
Position	Domain	Mutation
444	I (H25)	Transition (U→C)
985	I (H24)	Transition (U→C)
2392	V (H73)	Transition (C→U)

Supplementary Table 1 Mutations identified in the suppressor clones

The rDNA inserts of the plasmids recovered from two independent suppressor clones selected in the genetic screen (Sup. #2 and Sup. #10) were sequenced. The mutations identified in each case are detailed in the table: type of mutation, nucleotide position in the 25S rRNA sequence and corresponding structural domain. A C-to-U transition at position C2392 in helix H73 of the 25S rRNA (red on yellow background) has been identified in the two independent clones.

Strain	Doubling time (Minutes)
BY4741 (wild-type)	89 ± 0.3
BY4741 *snr190-[mut.C]*	102 ± 0.3
W303 (wild-type)	82
W303 *snr190-[mut.C]*	93 ± 2
**BY4741 *snr190-[mut.C]* + ...**	102 ± 0.3
E.V.	100.8
SNR190-U14 (wild-type)	87 ± 0.3
snr190-[mut.A]	86 ± 1
snr190-[mut.B]	85 ± 0.3
snr190-[mut.AB]	92 ± 0.3
**W303 *snr190-[mut.C]* + ...**	93 ± 2
E.V.	97
SNR190-U14 (wild-type)	85
snr190-[mut.A]	89.1
snr190-[mut.B]	88
snr190-[mut.AB]	93.3 ± 1
dbp7Δ	193
dbp7Δ , snr190-[mut.C]	157
dbp7Δ , snr190-[mut.A]	141 ± 2
dbp7Δ , snr190-[mut.G34C]	152 ± 2

Supplementary Table 2 Doubling time of yeast strain analyzed in this study

The indicated strains were grown under exponential conditions in YPD medium supplemented or not with G418 depending on the presence of pCH32 plasmid derivatives (G418 resistance marker). Turbidity at 600 nm was measured every two hours and the values were plotted over time in Excel with a logarithmic scale on the y axis. Doubling times were calculated according to the slopes of the curves.

STRAIN	BACKGROUND	PLASMID	GENOTYPE
**BY4741**	ø	ø	*MATa; his3Δ1; leu2Δ0; met15Δ0; ura3Δ0*
**W303**	ø	ø	*MATa; leu2-3,112; trp1-1; can1-100; ura3-1; ade2-1; his3-11,15;* [phi$^+$]
NOP7::HTP	BY4741	ø	*MATa; his3Δ1; leu2Δ0; met15Δ0; ura3Δ0; NOP7::HTP (URA3)*
NOP7::HTP, dbp7Δ	BY4741	ø	*MATa; his3Δ1; leu2Δ0; met15Δ0; ura3Δ0; dbp7::Kan^{R}MX6; NOP7::HTP (URA3)*
NOC1::TAP	BY4741	ø	*MATa; his3Δ1; leu2Δ0; met15Δ0; ura3Δ0; NOC1::TAP (HIS3)*
NOC1::TAP, snr190-[mut.C]	BY4741	ø	*MATa; his3Δ1; leu2Δ0; met15Δ0; ura3Δ0; snr190-[mut.C]* (CRISPR-Cas9); *NOC1::TAP (HIS3)*
NOC1::TAP, dbp7Δ	BY4741	ø	*MATa; his3Δ1; leu2Δ0; met15Δ0; ura3Δ0; dbp7::Kan^{R}MX6; NOC1::TAP (HIS3)*
**NOY891**	NOY505	pNOY353	*MATa; ade2-1; ura3-1; leu2-3; his3-11; trp1; can1-100; rdnΔΔ::HIS3; pNOY353 [PGAL7-35S rDNA, 5S rDNA (TRP1)]*
**EMY65**	NOY891	pNOY353	*MATa; ade2-1; ura3-1; leu2-3; his3-11; trp1; can1-100; rdnΔΔ::HIS3; GAL1::HA-DBP7 pNOY353 [PGAL7-35S rDNA, 5S rDNA(TRP1)]*
**EMY65 + pNOY373**	NOY891	pNOY353, pNOY373	*MATa; ade2-1; ura3-1; leu2-3; his3-11; trp1; can1-100; rdnΔΔ::HIS3; GAL1::HA-DBP7 pNOY353 [PGAL7-35S rDNA, 5S rDNA (TRP1)]; pNOY373 [35S rDNA, 5S rDNA (LEU2)]*
**EMY65 + pNOY373 C2392T**	NOY891	pNOY353, pNOY373 C2392T	*MATa; ade2-1; ura3-1; leu2-3; his3-11; trp1; can1-100; rdnΔΔ::HIS3; GAL1::HA-DBP7 pNOY353 [PGAL7-35S rDNA, 5S rDNA (TRP1)]; pNOY373 [35S rDNA, 5S rDNA (LEU2)]*
**EMY65 + Sup. #2**	NOY891	pNOY353, Sup. #2	*MATa; ade2-1; ura3-1; leu2-3; his3-11; trp1; can1-100; rdnΔΔ::HIS3; GAL1::HA-DBP7 pNOY353 [PGAL7-35S rDNA, 5S rDNA (TRP1)]; Sup. #2*
**EMY65 + Sup. #2 T2392C**	NOY891	pNOY353, Sup. #2 T2392C	*MATa; ade2-1; ura3-1; leu2-3; his3-11; trp1; can1-100; rdnΔΔ::HIS3; GAL1::HA-DBP7 pNOY353 [PGAL7-35S rDNA, 5S rDNA (TRP1)]; Sup. #2 T2392C*
**EMY65 + Sup. #10**	NOY891	pNOY353, Sup. #10	*MATa; ade2-1; ura3-1; leu2-3; his3-11; trp1; can1-100; rdnΔΔ::HIS3; GAL1::HA-DBP7 pNOY353 [PGAL7-35S rDNA, 5S rDNA (TRP1)]; Sup. #10*
**EMY65 + Sup. #10 T2392C**	NOY891	pNOY353, Sup. #10 T2392C	*MATa; ade2-1; ura3-1; leu2-3; his3-11; trp1; can1-100; rdnΔΔ::HIS3; GAL1::HA-DBP7 pNOY353 [PGAL7-35S rDNA, 5S rDNA (TRP1)]; Sup. #10 T2392C*
dbp7Δ	W303	ø	*MATa; ade2-1; ura3-1; leu2-3, 112; his3-11, 15; trp1-1; can1-100; dbp7::HIS3MX6*
dbp7Δ + DBP7	W303	*YCplac22-DBP7*	*MATa; ade2-1; ura3-1; leu2-3, 112; his3-11, 15; trp1-1; can1-100; dbp7::HIS3MX6;*
dbp7Δ + dbp7$_{K197A}$	W303	*YCplac22-dbp7-[K197A]*	*MATa; ade2-1; ura3-1; leu2-3, 112; his3-11, 15; trp1-1; can1-100; dbp7::HIS3MX6;*
W303 + E.V.	W303	*YCplac22*	*MATa; leu2-3,112; trp1-1; can1-100; ura3-1; ade2-1; his3-11,15;* [phi$^+$];
snr190-[mut.C] + E.V.	W303	*YCplac22*	*MATa; ade2-1; ura3-1; leu2-3, 112; his3-11, 15; trp1-1; can1-100; snr190-[mut.C];*
dbp7Δ + E.V.	W303	*YCplac22*	*MATa; ade2-1; ura3-1; leu2-3, 112; his3-11, 15; trp1-1; can1-100; dbp7::HIS3MX6;*
dbp7Δ snr190-[mut.C] + E.V.	W303	*YCplac22*	*MATa; ade2-1; ura3-1; leu2-3, 112; his3-11, 15; trp1-1; can1-100; dbp7::HIS3MX6 snr190-[mut.C];*
dbp7Δ snr190-[mut.C] + dbp7$_{K197A}$	W303	*YCplac22-dbp7-[K197A]*	*MATa; ade2-1; ura3-1; leu2-3, 112; his3-11, 15; trp1-1; can1-100; dbp7::HIS3MX6 snr190-[mut.C];*
dbp7Δ snr190-[mut.C] + DBP7	W303	*YCplac22-DBP7*	*MATa; ade2-1; ura3-1; leu2-3, 112; his3-11, 15; trp1-1; can1-100; dbp7::HIS3MX6 snr190-[mut.C];*
dbp7Δ , snr190-[mut.C]	BY4741	ø	*MATa; his3Δ1; leu2Δ0; met15Δ0; ura3Δ0; dbp7::Kan^{R}MX6; snr190-[mut.C]* (CRISPR-Cas9)
dbp7Δ , snr190-[mut.A]	BY4741	ø	*MATa; his3Δ1; leu2Δ0; met15Δ0; ura3Δ0; dbp7::Kan^{R}MX6; snr190-[mut.A]* (CRISPR-Cas9)
dbp7Δ , snr190-[mut.G34C]	BY4741	ø	*MATa; his3Δ1; leu2Δ0; met15Δ0; ura3Δ0; dbp7::Kan^{R}MX6; snr190-[mut.G34C]* (CRISPR-Cas9)
snr190-[mut.C]	BY4741	ø	*MATa; his3Δ1; leu2Δ0; met15Δ0; ura3Δ0; snr190-[mut.C]* (CRISPR-Cas9)
snr190-[mut.C] + E.V.	BY4741	pCH32	*MATa; his3Δ1; leu2Δ0; met15Δ0; ura3Δ0; snr190-[mut.C]* (CRISPR-Cas9)
snr190-[mut.C] + SNR190	BY4741	pCH32 + WT *U14-SNR190* insert	*MATa; his3Δ1; leu2Δ0; met15Δ0; ura3Δ0; snr190-[mut.C]* (CRISPR-Cas9)
snr190-[mut.C] + snr190-[mut.A]	BY4741	pCH32 + *U14-snr190-[mut.A]*	*MATa; his3Δ1; leu2Δ0; met15Δ0; ura3Δ0; snr190-[mut.C]* (CRISPR-Cas9)
snr190-[mut.C + snr190-[mut.B]	BY4741	pCH32 + *U14-snr190-[mut.B]*	*MATa; his3Δ1; leu2Δ0; met15Δ0; ura3Δ0; snr190-[mut.C]* (CRISPR-Cas9)
snr190-[mut.C] + snr190-[mut.AB]	BY4741	pCH32 + *U14-snr190-[mut.AB]*	*MATa; his3Δ1; leu2Δ0; met15Δ0; ura3Δ0; snr190-[mut.C]* (CRISPR-Cas9)
snr190-[mut.C] + snr190-[mut.S]	BY4741	pCH32 + *U14-snr190-[mut.S]*	*MATa; his3Δ1; leu2Δ0; met15Δ0; ura3Δ0; snr190-[mut.C]* (CRISPR-Cas9)
snr190-[mut.C]	W303	ø	*MATa; leu2-3,112; trp1-1; can1-100; ura3-1; ade2-1; his3-11,15;* [phi$^+$]; *snr190-[mut.C]* (CRISPR-Cas9)
snr190-[mut.C] + E.V.	W303	pCH32	*MATa; leu2-3,112; trp1-1; can1-100; ura3-1; ade2-1; his3-11,15;* [phi$^+$]; *snr190-[mut.C]* (CRISPR-Cas9)
snr190-[mut.C] + SNR190	W303	pCH32 + WT *U14-SNR190* insert	*MATa; leu2-3,112; trp1-1; can1-100; ura3-1; ade2-1; his3-11,15;* [phi$^+$]; *snr190-[mut.C]* (CRISPR-Cas9)
snr190-[mut.C] + snr190-[mut.A]	W303	pCH32 + *U14-snr190-[mut.A]*	*MATa; leu2-3,112; trp1-1; can1-100; ura3-1; ade2-1; his3-11,15;* [phi$^+$]; *snr190-[mut.C]* (CRISPR-Cas9)
snr190-[mut.C] + snr190-[mut.B]	W303	pCH32 + *U14-snr190-[mut.B]*	*MATa; leu2-3,112; trp1-1; can1-100; ura3-1; ade2-1; his3-11,15;* [phi$^+$]; *snr190-[mut.C]* (CRISPR-Cas9)
snr190-[mut.C + snr190-[mut.AB]	W303	pCH32 + *U14-snr190-[mut.AB]*	*MATa; leu2-3,112; trp1-1; can1-100; ura3-1; ade2-1; his3-11,15;* [phi$^+$]; *snr190-[mut.C]* (CRISPR-Cas9)

Supplementary Table 3 Yeast strains used in this study

Inventory of the yeast strains used in this study with their genetic background (W303 or BY4741), the enclosed plasmids and genotypes.

OLIGONUCLEOTIDE	SEQUENCE (5'- 3')	EXPERIMENT
OHA494	GATCAAGAGACACCATTATCATCAGTTTTAGAGCTAG	Mutagenesis of *SNR190* box C by CRISPR-Cas9, cloning into pML104
OHA495	CTAGCTCTAAAACTGATGATAATGGTGTCTCTT	Mutagenesis of SNR190 box C by CRISPR-Cas9, cloning into pML104
OHA496	GAAATTTCATGACACTTTTTTAAACAGAAAAGAAGAATTGATTGGAACTATTGATAATGGTGTCTCTTCTTTCCTCGTCCGATTCGACCATGACGACAA	Mutagenesis of *SNR190* box C by CRISPR-Cas9, donnor oligonucleotide
OHA504	GATCCGTCATGGTCGAATCGGACGGTTTTAGAGCTAG	Mutagenesis of *SNR190* box A by CRISPR-Cas9, cloning into pML104
OHA505	CTAGCTCTAAAACCGTCCGATTCGACCATGACG	Mutagenesis of *SNR190* box A by CRISPR-Cas9, cloning into pML104
OHA506	AAACAGAAAAGAAGAATTGATTGGCCCTGATGATAATGGTGTCTGTTATTTGAAGATCCGATTCGACCATGACGACAAGGGATTTTATCTCGTTCTCTT	Mutagenesis of *SNR190* box A by CRISPR-Cas9, donnor oligonucleotide
OMJ132	AAAAGAAGAATTGATTGGCCCTGATGATAATGGTGTCTCTTCTTTACTCCTCCGATTCGACCATGACGACAAGGGATTTTATCTCGTTCTCTTAATGCG	Mutagenesis of *SNR190* box A, G34C, by CRISPR-Cas9, donnor oligonucleotide
OMJ122	GACGGCCAGTGAATTCATCAACTCCCCTTCAAAT	Genomic amplification of *U14-SNR190* gene
OMJ123	AAGTTATTAGGTGATATCTATCAATTTTTTGTTAATCCCGT	Genomic amplification of *U14-SNR190* gene
OMJ124	GATCTTCAAATAACAGACACCATTATCATCAGGGCC	Mutagenesis of snR190 box A on plasmid *pCH32-U14-SNR190* by InFusion
OMJ125	TGTTATTTGAAGATCCGATTCGACCATGACG	Mutagenesis of snR190 box A on plasmid *pCH32-U14-SNR190* by InFusion
OMJ126	TAAGTGGCCGAACAAAAGTTCCCGTTTGCAAACAC	Mutagenesis of snR190 box B on plasmid *pCH32-U14-SNR190* by InFusion
OMJ127	TTGTTCGGCCACTTATACAACACATGCAGATCTGAGCC	Mutagenesis of snR190 box B on plasmid *pCH32-U14-SNR190* by InFusion
OMJ138	TAAAAGGTTGTTTCGTCATGGTCGAATCGGAC	Mutagenesis of snR190 internal stem on plasmid *pCH32-U14-SNR190* by InFusion
OMJ139	CGAAACAACCTTTTATCTCGTTCTCTTAATGCGA	Mutagenesis of snR190 internal stem on plasmid *pCH32-U14-SNR190* by InFusion
OHA497BIS	GCCAAGCGCGCAATTAACCC	pML104 sequencing for mutagenesis by CRISPR-Cas9
OHA497	CGACTTCAGCATTGCACTCC	Genomic *SNR190* sequencing
OHA498	CCGAGAGTACTAACGATGGG	Genomic *SNR190* sequencing
OHA602	AGAATCAGTGGGGAAAGAAGACCCTGTTGAG	Mutagenesis of 25S rRNA C2392U on plasmid *pNOY373* by InFusion
OHA603	TTCCCCACTGATTCTGCCAAGCCCGTTC	Mutagenesis of 25S rRNA C2392U on plasmid *pNOY373* by InFusion
OHA604	AGAATCAGCGGGGAAAGAAGACCCTGTTGAG	Mutagenesis of 25S rRNA U2392C on plasmids *Sup. #2* and *Sup. #10* by InFusion
OHA605	TTCCCCGCTGATTCTGCCAAGCCCGTTC	Mutagenesis of 25S rRNA U2392C on plasmids *Sup. #2* and *Sup. #10* by InFusion
OHA606	CTTAGAACTGGTACGGACAAGG	Amplification and sequencing of 25S rDNA on *pNOY373*
OHA607	TCGATAGGCCACACTTTCATGG	Amplification and sequencing of 25S rDNA on *pNOY373*
RT Primer #1	TACACCCTCTATGTCTCTTCAC	OMJ147, methylation mapping by RT, 25S rRNA position 2434 to 2455
RT Primer #2	CACAATGTCAAACTAGAGTCAAGC	OMJ146, methylation mapping by RT, 25S rRNA position 2413 to 2436
Nop7-HTP-Fwd	ATTGCCAAACAAAAAGCTAAACTGAATAAACTAGATTCCAAGAAAGAGCACCATCACCATCACC	*NOP7::HTP* strain construction
Nop7-HTP-Rev	TAAAGTAATAAAGATGTTAAATTAAAAGACAAAATTTTTGAGAGGTACGACTCACTATAGGG	*NOP7::HTP* strain construction
Noc1-TAP-Fwd	ACGACAGTGAACTTGACTTCGC	*NOC1::TAP* strain construction
Noc1-TAP-Rev	GATGACTTGAAGAGCGCATTCG	*NOC1::TAP* strain construction
23S.1	GATTGCTCGAATGCCCAAAG	Northern blotting probe, detection of 35S, 33S/32S, 27SA2 and 23S pre-rRNAs
rRNA2.1	GGCCAGCAATTTCAAGTTA	Northern blotting probe, detection of 35S, 33S/32S, 27SA2, 27SB and 7S pre-rRNAs
20S.3	TTAAGCGCAGGCCCGGCTGG	Northern blotting probe, detection of 35S, 33S/32S, 23S and 20S pre-rRNAs
25S	CCATCTCCGGATAAACC	Northern blotting probe, detection of mature 25S rRNA
18S middle	CATGGCTTAATCTTTGAGAC	Northern blotting probe, detection of mature 18S rRNA
5.8S	TGCGTTCAAAGATTCGATG	Northern blotting probe, detection of mature 5.8S rRNAs
5S	CATCACCCACTGGTATGCGC	Northern blotting probe, detection of mature 5S rRNA
SNR190 #1	CCTTGTCGTCATGGTCGAATCG	Northern blotting probe, detection of snR190 snoRNA (nucleosides 37 to 58)
SNR190 #2	CGAGGAAAGAAGAGACACCATTATC	Northern blotting probe, detection of snR190 snoRNA (nucleosides 10 to 34)
U14	CTCAGACATCCTAGGAAGG	Northern blotting probe, detection of U14 snoRNA (nucleosides 105 to 123)
SNR37	GATAGTATTAACCACTACTG	Northern blotting probe, detection of snR37 snoRNA (nucleosides 6 to 25)
SNR38	CCATTCAGACAGGGATAACTGTTTG	Northern blotting probe, detection of snR38 snoRNA (nucleosides 25 to 49)
SNR71	GATCTGAGTGAGCTGAGAAGGTT	Northern blotting probe, detection of snR71 snoRNA (nucleosides 14 to 36)
SNR5	GTCTACTTCCAGCCATTTGC	Northern blotting probe, detection of snR5 snoRNA (nucleosides 71 to 90)
SNR10	CATGGGTCAAGAACGCCCCGGAGGGG	Northern blotting probe, detection of snR10 snoRNA (nucleosides 54 to 79)
SNR42	TCAAACAATAGGCTCCCTAAAGCATCACAA	Northern blotting probe, detection of snR42 snoRNA (nucleosides 9 to 38)
SNR61	GGTTCAGAAGCAGAACTGAATAG	Northern blotting probe, detection of snR61 snoRNA (nucleosides 22 to 44)

Supplementary Table 4 Oligonucleotides used in this study

Inventory and sequence of the oligonucleotides used in this study.

SUPPLEMENTARY REFERENCES

1. Cannone, J. J. *et al.* The comparative RNA web (CRW) site: an online database of comparative sequence and structure information for ribosomal, intron, and other RNAs. *BMC Bioinformatics* **3,** 2 (2002).

2. Lindahl, L. *et al.* RNase MRP is required for entry of 35S precursor rRNA into the canonical processing pathway. *RNA* **15,** 1407–1416 (2009).

3. de la Cruz, J., Kressler, D., Rojo, M., Tollervey, D. & Linder, P. Spb4p, an essential putative RNA helicase, is required for a late step in the assembly of 60S ribosomal subunits in *Saccharomyces cerevisiae*. *RNA* **4,** 1268–1281 (1998).

4. Rosado, I. V. *et al.* Characterization of *Saccharomyces cerevisiae* Npa2p (Urb2p) reveals a low-molecular-mass complex containing Dbp6p, Npa1p (Urb1p), Nop8p, and Rsa3p involved in early steps of 60S ribosomal subunit biogenesis. *Mol. Cell. Biol.* **27,** 1207–1221 (2007).
