# Supplementary material for: Association of snR190 snoRNA chaperone with early pre-60S particles is regulated by the RNA helicase Dbp7 in yeast
Source: Nat Commun. 2021 Oct 22;12:6153. doi: 10.1038/s41467-021-26207-w (PMC8536666; doi:10.1038/s41467-021-26207-w)
Supplement: Supplementary file 3 — Reporting summary [file 41467_2021_26207_MOESM3_ESM.pdf]

nature research

Corresponding author(s): Anthony K. Henras

Last updated by author(s): Sep 1, 2021

Reporting Summary

Nature Research wishes to improve the reproducibility of the work that we publish. This form provides structure for consistency and transparency in reporting. For further information on Nature Research policies, see our Editorial Policies and the Editorial Policy Checklist.

Statistics

For all statistical analyses, confirm that the following items are present in the figure legend, table legend, main text, or Methods section.

n/a	Confirmed	
[ ]	[x]	The exact sample size (*n*) for each experimental group/condition, given as a discrete number and unit of measurement
[ ]	[x]	A statement on whether measurements were taken from distinct samples or whether the same sample was measured repeatedly
[ ]	[x]	The statistical test(s) used AND whether they are one- or two-sided<br>*Only common tests should be described solely by name; describe more complex techniques in the Methods section.*
[x]	[ ]	A description of all covariates tested
[x]	[ ]	A description of any assumptions or corrections, such as tests of normality and adjustment for multiple comparisons
[ ]	[x]	A full description of the statistical parameters including central tendency (e.g. means) or other basic estimates (e.g. regression coefficient) AND variation (e.g. standard deviation) or associated estimates of uncertainty (e.g. confidence intervals)
[ ]	[x]	For null hypothesis testing, the test statistic (e.g. *F*, *t*, *r*) with confidence intervals, effect sizes, degrees of freedom and *P* value noted<br>*Give P values as exact values whenever suitable.*
[x]	[ ]	For Bayesian analysis, information on the choice of priors and Markov chain Monte Carlo settings
[x]	[ ]	For hierarchical and complex designs, identification of the appropriate level for tests and full reporting of outcomes
[x]	[ ]	Estimates of effect sizes (e.g. Cohen's *d*, Pearson's *r*), indicating how they were calculated

Our web collection on statistics for biologists contains articles on many of the points above.

Software and code

Policy information about availability of computer code

Data collection	Western blot data were collected using a ChemiDoc Touch Imaging System (Bio-Rad) and Image Lab software (Version 5.2.1, Bio-Rad).<br>Radioactive northern blotting data were revealed from PhosphorImager screens (FUJIFILM) using Typhoon TRIO or Typhoon 9400 Variable Mode Imagers (GE Healthcare) driven by Typhoon Scanner Control software (Version 5.0).<br>Sucrose gradient fractions were collected using a Foxy R1 gradient collection system (TELEDYNE ISCO) driven by PeakTrak software (Version 1.10, Isco Inc.).The A254 absorbance profiles were measured during collection with a UA-6 UV/VIS DETECTOR (Teledyne Isco).<br>Oligonucleotides for the CRISPR-Cas9 approach were designed using the online CRISPR Toolset (http://wyrickbioinfo2.smb.wsu.edu/crispr.html).
Data analysis	For western blot data, ChemiDoc Touch images were quantified using Image Lab software (Version 5.2.1, Bio-Rad).<br>For northern blot data, PhosphorImager images were quantified using Multi Gauge software (Version 3.0, FUJIFILM).<br>For polysome profiles, PeakTrak data were converted to .txt files and used in Excel (Version 2016) to generate the profiles displayed in the figures.

For manuscripts utilizing custom algorithms or software that are central to the research but not yet described in published literature, software must be made available to editors and reviewers. We strongly encourage code deposition in a community repository (e.g. GitHub). See the Nature Research guidelines for submitting code & software for further information.

Data

Policy information about availability of data

All manuscripts must include a data availability statement. This statement should provide the following information, where applicable:

- Accession codes, unique identifiers, or web links for publicly available datasets
- A list of figures that have associated raw data
- A description of any restrictions on data availability

The datasets generated during and/or analysed during the current study are available in the supplementary material.
Any other information can be obtained from the corresponding author on reasonable request.

Field-specific reporting

Please select the one below that is the best fit for your research. If you are not sure, read the appropriate sections before making your selection.

[x] Life sciences [ ] Behavioural & social sciences [ ] Ecological, evolutionary & environmental sciences

For a reference copy of the document with all sections, see nature.com/documents/nr-reporting-summary-flat.pdf

Life sciences study design

All studies must disclose on these points even when the disclosure is negative.

Sample size	The most important conclusions of this study were infered from the analysis of two independent yeast mutant strains lacking snR190 or bearing mutations in its functional regions in two different genetic backgrounds (W303 and BY4741). These four biological replicates were analyzed in at least two independent experiments (at least 2 technical replicates).
Data exclusions	No data were excluded from the analyses.
Replication	The suppressor mutation at the basis of the study was first identified in two independent suppressor mutants. The phenotypic features of this mutation were then independently reassessed by introducing or removing this mutation from the expression plasmid by site-directed mutagenesis and consistent results were obtained (Figure 1).<br>For northern blotting experiments , reproducibility was granted by the fact that all the mutant strains within a given group of biological replicates all showed similar phenotypes that differed from the corresponding wild-type strains. Most quantifications were performed out of four replicates (two biological replicates analyzed in two technical replicates) in two different genetic backgrounds (W303 and BY4741).<br>The retention of snR190 and other snoRNAs in pre-60S particles upon inactivation of Dbp7 was analyzed using two different means of inactivating Dbp7 (full depletion or expression of a catalytic mutant) and two different methodologies: co-immunoprecipitations (Figure 5a, b) and sucrose gradient sedimentation experiments (Figures 5c and 6c).
Randomization	Allocation of samples into experimental groups was not random. All mutant strains in a given genetic background (BY4741 or W303) were compared among themselves and with the corresponding wild-type strain because of significant variations in the phenotypic features of ribosome biogenesis depending on the genetic background.
Blinding	Blinding was not relevant to this study. All values used to quantify the data were obtained from image analysis softwares (Multi Gauge, Image Lab) which do not rely on the assessment of the investigators and cannot be biased by the investigators.

Reporting for specific materials, systems and methods

We require information from authors about some types of materials, experimental systems and methods used in many studies. Here, indicate whether each material, system or method listed is relevant to your study. If you are not sure if a list item applies to your research, read the appropriate section before selecting a response.

Materials & experimental systems

n/a	Involved in the study
[ ]	[x] Antibodies
[ ]	[x] Eukaryotic cell lines
[x]	[ ] Palaeontology and archaeology
[x]	[ ] Animals and other organisms
[x]	[ ] Human research participants
[x]	[ ] Clinical data
[x]	[ ] Dual use research of concern

Methods

n/a	Involved in the study
[x]	[ ] ChIP-seq
[x]	[ ] Flow cytometry
[x]	[ ] MRI-based neuroimaging

Antibodies

Antibodies used	Anti-Prp43 primary antibodies, 1:1000 dilution (Henry/Henras laboratory);<br>Anti-Nhp2 primary antibodies, 1:1000 dilution (Henry/Henras laboratory);<br>Anti-Npa1 primary antibodies, 1:5000 dilution (Henry/Henras laboratory);<br>Anti-Dbp6 primary antibodies, 1:10 000 dilution (Henry/Henras laboratory);<br>Anti-Nop8 primary antibodies, 1:2000 dilution (Henry/Henras laboratory);<br>Anti-Rsa3 primary antibodies, 1:10 000 dilution (Henry/Henras laboratory);<br>Anti-Pgk1 primary antibodies, Clone 1086CT10.2.1, 1:8000 dilution (Invitrogen, Cat. # MA5-37712);<br>Anti-HA-Peroxidase, High Affinity (3F10) antibodies, 1:1000 dilution (Roche Diagnostics, Cat. #12013819001);<br>HRP-conjugated anti-Mouse antibodies, 1:10 000 dilution (Promega, W402B, Lot #0000346265)<br>HRP-conjugated anti-Rabbit antibodies, 1:10 000 dilution (Promega, W401B, Lot #0000407624)
Validation	Anti-Prp43 primary antibodies, custom production: Lebaron et al., 2005, MCB (doi: 10.1128/MCB.25.21.9269-9282.2005);<br>Anti-Nhp2 primary antibodies, custom production: Henras et al., 2001, NAR (doi: 10.1093/nar/29.13.2733);<br>Anti-Npa1 primary antibodies, custom production: Joret et al., 2018, PLoS Genet. (doi: 10.1371/journal.pgen.1007597);<br>Anti-Dbp6 primary antibodies, custom production: Joret et al., 2018, PLoS Genet. (doi: 10.1371/journal.pgen.1007597);<br>Anti-Nop8 primary antibodies, custom production: Joret et al., 2018, PLoS Genet. (doi: 10.1371/journal.pgen.1007597);<br>Anti-Rsa3 primary antibodies, custom production: Joret et al., 2018, PLoS Genet. (doi: 10.1371/journal.pgen.1007597);<br>Anti-Pgk1 primary antibodies: please refer to the Source Data file: a single yeast protein at the size expected for yeast Pgk1 (about 45 kDa) is detected with these antidodies on total yeast extracts.<br>Anti-HA-Peroxidase antibodies: please refer to the Source Data file: a single yeast protein at the size expected for Dbp7-HA (about 100 kDa) is detected with these antidodies in extracts containing Dbp7-HA but not in control extracts without HA-tagged proteins.

Eukaryotic cell lines

Policy information about cell lines

Cell line source(s)	All yeast strains used in this study were derivatives of either Saccharomyces cerevisiae strains BY4741 or W303.<br>These strains are listed and described in the Supplementary Table 3.
Authentication	Yeast strains were authenticated on the basis of the selection markers.<br>CRISPR-Cas9-based genome editing was verified by sequencing of PCR-amplified genomic loci.<br>Mutations introduced on plasmids using the InFusion method were verified by sequencing before transformation of the plasmids into yeast cells.
Mycoplasma contamination	n/a
Commonly misidentified lines<br>(See ICLAC register)	n/a
